# A Hyaluronan-binding Peptide (P15-1) Reduces Inflammatory and Catabolic Events in IL-1β-treated Human Articular Chondrocytes

**DOI:** 10.1038/s41598-020-57586-7

**Published:** 2020-01-29

**Authors:** Claire Shortt, Leonard G. Luyt, Eva A. Turley, Mary K. Cowman, Thorsten Kirsch

**Affiliations:** 1 Musculoskeletal Research Center, Department of Orthopaedic Surgery, New York University School of Medicine, New York, NY USA; 2 The University of Western Ontario, London, ON Canada; 3 London Regional Cancer Program, Lawson Health Research Institute, London, ON Canada; 4 Department of Biomedical Engineering, New York University Tandon School of Engineering, New York, NY USA; 5 Present Address: FoodMarble Digestive Health, Dublin, 2 Ireland

**Keywords:** Mechanisms of disease, Cartilage

## Abstract

Inflammation plays a critical role in osteoarthritis (OA). It stimulates catabolic events in articular chondrocytes and prevents chondrogenic precursor cells from repairing cartilage lesions, leading to accelerated cartilage degradation. Therefore, the identification of novel factors that reduce catabolic events in chondrocytes and enhances chondrogenic differentiation of precursor cells in an inflammatory environment may provide novel therapeutic strategies for the treatment of OA. The goal of this study was to determine whether a hyaluronan (HA)-binding peptide (P15-1), via interacting with high molecular weight (HMW)HA can enhance the anti-inflammatory properties of HMWHA and decrease catabolic events in interleukin-1beta (IL-1β)-treated human articular chondrocytes. Treatment with P15-1 decreased catabolic events and stimulated anabolic events in articular chondrocytes cultured in an inflammatory environment. P15-1 pre-mixed with HMWHA was more effective in inhibiting catabolic events and stimulating anabolic events than P15-1 or HMWHA alone. Our findings suggest that P15-1 together with HMWHA inhibits catabolic events in articular chondrocytes via the inhibition of p38 mitogen-activated protein kinases (MAPK) and increasing the thickness of the pericellular matrix (PCM) around chondrocytes thereby decreasing catabolic signaling. Finally, conditioned medium from IL-1β and P15-1-treated human articular chondrocytes was less inhibitory for chondrogenic differentiation of precursor cells than conditioned medium from chondrocytes treated with IL-1β alone. In conclusion, P15-1 is proposed to function synergistically with HMWHA to enhance the protective microenvironment for chondrocytes and mesenchymal stem cells during inflammation and regeneration.

## Introduction

Articular cartilage is vulnerable to multifactorial damage and its insufficiency to self-repair leads to progressive pain and functional limitation over time. In addition, the repair of cartilage lesions is made more difficult because the chondrocytes surrounding the lesions normally show high catabolic activity in osteoarthritis (OA) or after injury. This high catabolic activity leads to the release of cytokines, factors and proteases that inhibit chondrogenic differentiation of precursor cells present in the joint and ultimately the formation of hyaline-like cartilage repair tissue in the lesion site^1^. The production and activities of many pro-inflammatory factors are controlled by different signaling systems, such as nuclear factor-kappa B (NF-κB), mitogen-activated protein kinases (MAPK) and Janus kinase/signal transducers and activators of transcription (JAK/STAT)^2–4^.

Hyaluronan (HA), a member of the glycosaminoglycan (GAG) family, which is a main component of the extracellular matrix (ECM), is a crucial component of the pericellular matrix (PCM) of mesenchymal stem cells (MSCs) and chondrocytes^5,6^. HA is considered to be an essential component of the stem cell niche and a suppressor of inflammation^5,7–9^. HA is composed of alternating β-D-glucuronic acid and N-acetyl-β-D-glucosamine residues. This molecular unit repeated thousands of times forms a very long linear polymer with molecular weight reaching 8 × 10^6^ Da^10,11^. HA can interact with several receptors, with CD44 being the most prominent receptor interacting with high molecular weight (HMW)HA^12^. HMWHA binds to CD44 and clusters CD44^13^. Clustering of CD44 may make this receptor unavailable to act as a co-receptor for other receptors, including toll-like receptors (TLRs), which upon ligand binding initiate catabolic signaling in chondrocytes^13,14^. In addition, it has been suggested that HMWHA provides a coat around the cell, which is able to mask receptors, such as TLRs and other receptors that mediate catabolic signaling and subsequently prevent the stimulation of these receptors^11,15^.

HMWHA has been shown to inhibit catabolic signaling, including NF-κB and p38 MAPK signaling^9,16,17^. Especially, the p38 MAPK signaling pathway has been shown to be correlated with inflammation in OA^17,18^. Inflammatory cytokines, such as interleukin-1beta (IL-1β) activate p38 MAPK signaling leading to the expression of IL-1β-induced catabolic mediators, such as interleukin-6 (IL-6) in OA cartilage^19^. Consequently, inhibition of p38 MAPK signaling pathway has been shown to decrease the expression of inflammatory cytokines in articular chondrocytes^20^. A previous study has shown that HA reduced IL-1β-induced MMP-13 expression via the inhibition of p38 MAPK signaling^16^.

Maintaining HA integrity or restoring HA size and integrity in the extracellular matrix of articular cartilage may resolve inflammation and catabolism in articular cartilage and ultimately may help to preserve and repair cartilage. Recently, our collaborators discovered a 15-mer peptide (P15-1) that binds to HA and showed that this peptide reduced inflammation and fibrogenesis in excisional skin wounds^21^. Therefore, the goal of this study was to determine whether P15-1, via interacting with HMWHA, is able to protect chondrocytes against the stimulation of catabolic events by an inflammatory environment as present in injured joints or joints affected by osteoarthritis (OA). To achieve this goal, we cultured human articular chondrocytes under inflammatory conditions (in the presence of IL-1β) and determined how P15-1 together with HMWHA affects catabolic events in these cells. Furthermore, we determined whether P15-1 together with HMWHA can enhance chondrogenic differentiation of precursor cells when cultured in an inflammatory environment.

## Results

### P15-1 decreased catabolic and inflammatory events in human articular chondrocytes

Even though human articular chondrocytes were isolated from cartilage samples showing no evident signs of macroscopical degradation from patients undergoing knee replacement surgery, these cells showed some expression levels of catabolic markers (cyclooxygenase-2 (Cox-2), IL-6, inducible nitric oxide synthase (iNOS), matrix metalloproteinase-13 (MMP-13)) when cultured under serum-free conditions for 24 h. IL-1β treatment of these cells, however, resulted in a marked increase of mRNA levels of these catabolic markers relative to the levels of vehicle-treated cells (between ~25-fold to ~1200-fold increased mRNA levels of these catabolic markers), and a decrease in the mRNA levels of articular cartilage (anabolic) markers, including aggrecan and type II collagen (between ~60% to 80% decreased mRNA levels of cartilage markers) relative to the levels of vehicle-treated cells (Suppl. Fig. 1).

**Figure 1:**
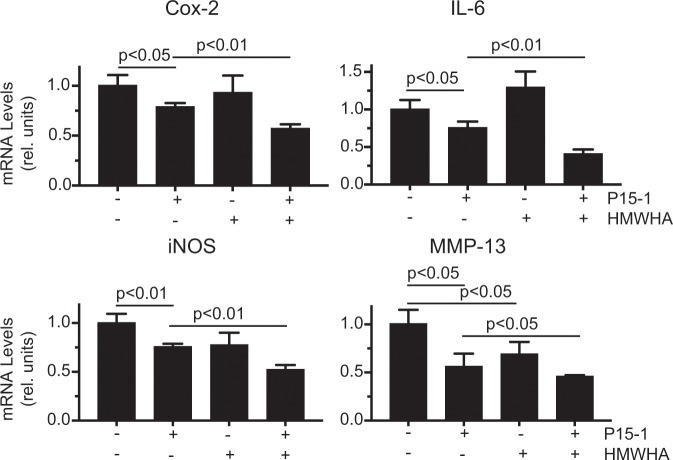
mRNA levels of catabolic markers in human articular chondrocytes cultured in the absence or presence of P15-1 and HMWHA. Human articular chondrocytes after reaching confluency were serum-starved for 24 h followed by treatment with P15-1, HMWHA, or P15-1 and HMWHA for 24 h. mRNA levels of catabolic markers (Cox-2, IL-6, iNOS, MMP-13) were determined by real-time PCR using SYBR Green and normalized to the level of 18S RNA. The mRNA levels are expressed relative to the level of vehicle-treated cells, which was set as 1. Data were obtained from triplicate PCRs using RNA from 3 different cultures. Values are the mean ± SD.

**Figure 2:**
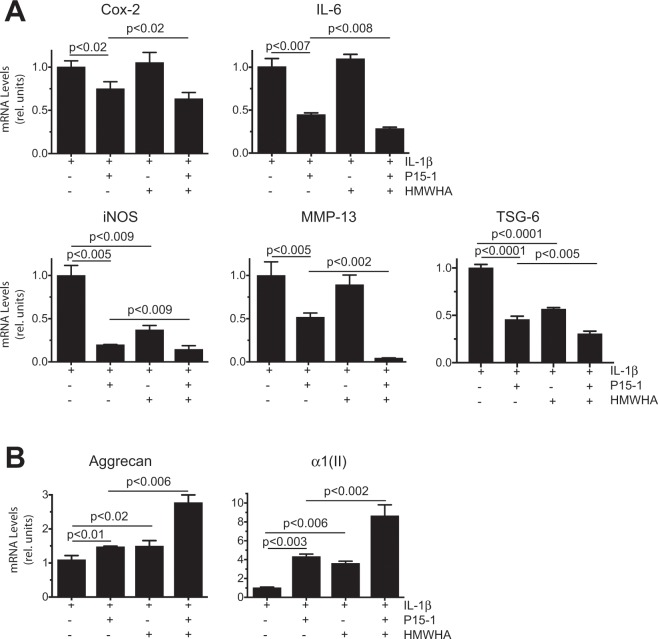
mRNA levels of catabolic markers and TSG-6 (**A**), and articular cartilage (anabolic) markers (**B**) in IL-1β-treated human articular chondrocytes cultured in the absence or presence of P15-1, and HMWHA. Human articular chondrocytes after reaching confluency were serum-starved for 24 h followed by treatment with IL-1β, IL-1β and P15-1, IL-1β and HMWHA, or IL-1β, P15-1 and HMWHA for 24 h. mRNA levels of (**A**) catabolic markers (Cox-2, IL-6, iNOS, MMP-13) and TSG-6, and (**B**) articular cartilage markers (aggrecan, type II collagen (α1(II)) were determined by real-time PCR using SYBR Green and normalized to the level of 18S RNA. The mRNA levels are expressed relative to the level of IL-1β - treated cells, which was set as 1. Data were obtained from triplicate PCRs using RNA from 3 different cultures. Values are the mean ± SD.

P15-1 alone reduced the mRNA levels of the catabolic markers Cox-2, IL-6, iNOS and MMP-13 in vehicle-treated and IL-1β-treated human articular chondrocytes to various degrees (Figs. 1 and 2A). In vehicle-treated cells, HMWHA alone reduced MMP-13 mRNA levels (Fig. 1). In IL-1β-treated chondrocytes, HMWHA alone reduced the mRNA levels of iNOS, but to a lesser degree than P15-1 (Fig. 2A). HMWHA did not significantly change the mRNA levels of Cox-2, IL-6 and iNOS mRNA levels in vehicle-treated cells, and the mRNA levels of Cox-2, IL-6 and MMP-13 in IL-1β-treated chondrocytes (Figs. 1 and 2A). When P15-1 was pre-mixed with HMWHA, and then the mixture was used to treat human articular chondrocytes in the absence or presence of IL-1β, P15-1 together with HMWHA synergistically resulted in a further decrease of the mRNA levels of Cox-2, IL-6, iNOS and MMP-13 compared to P15-1 alone or HMWHA alone (Figs. 1 and 2A). P15-1 in combination with HMWHA reduced the mRNA levels of the tested catabolic markers by ~40% and more in vehicle-treated and IL-1β-treated chondrocytes. In addition, the ratio of P15-1 to HMWHA of 1:60 as calculated based on the one P15-1 peptide per 75 binding sites on HMWHA was the most effective ratio to inhibit catabolic events and stimulate anabolic events in IL-1β-treated chondrocytes compared to other ratios (1:15, 1:30, 1:120) tested (data not shown).

Tumor necrosis factor-inducible gene 6 protein (TSG-6) is a protein that binds to HA and is able to cross-link HMWHA^22^. In addition, this protein is upregulated in many cell types in inflammatory disease states, including articular chondrocytes in OA^23–25^. Here we show that the expression of TSG-6 in vehicle-treated articular chondrocytes was not detectable by real time PCR analysis. However, TSG-6 expression was detectable in IL-1β-treated chondrocytes. P15-1 as well as HMWHA treatment markedly reduced the mRNA levels of TSG-6 in IL-1β-treated chondrocytes. P15-1 together with HMWHA was more effective in reducing the mRNA levels of TSG-6 in IL-1β-treated chondrocytes than P-15-1 or HMWHA alone (Fig. 2A).

Furthermore, P15-1 together with HMWHA was the most effective treatment increasing the mRNA levels of the articular cartilage markers aggrecan and type II collagen, whereas P15-1 alone or HMWHA alone were less effective increasing the mRNA levels of aggrecan and type II collagen. (Fig. 2B). P15-1, HMWHA, and P15-1 together with HMWHA, however, did not affect the mRNA levels of aggrecan and type II collagen in vehicle-treated chondrocytes (data not shown). These findings show that P15-1 reduced catabolic events in vehicle-treated and IL-1β-treated chondrocytes, and increased the mRNA levels of aggrecan and type II collagen only in IL-1β-treated human articular chondrocytes. P15-1 together with HMWH acted synergistically, and both together were more effective in inhibiting catabolic events in vehicle-treated and IL-1β-treated chondrocytes, and increasing articular cartilage markers (aggrecan, type II collagen) in Il-1β-treated human articular chondrocytes than P15-1 or HMWHA alone.

### P15-1 affects HA receptor CD44 and TLR receptor expression in IL-1β-treated human articular chondrocytes

**Figure 3:**
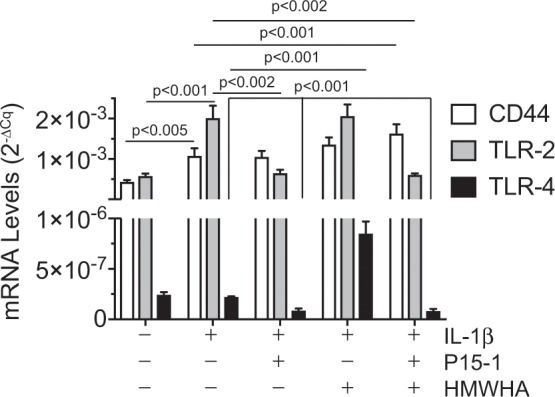
mRNA levels of CD44, TLR-2 and TLR-4 in human articular chondrocytes cultured in the absence or presence of IL-1β, P15-1, and HMWHA. Human articular chondrocytes after reaching confluency were serum-starved for 24 h followed by treatment with IL-1β, IL-1β and P15-1, IL-1β and HMWHA, or IL-1β, P15-1 and HMWHA for 24 h. mRNA levels of CD44, TLR-2 TLR-4 were determined by real-time PCR using SYBR Green and normalized to the level of 18S RNA. Data were obtained from triplicate PCRs using RNA from 3 different cultures. Values are the mean ± SD.

IL-1β treatment increased the mRNA levels of the main HA receptor, CD44 and the inflammatory receptor toll-like receptor-2 (TLR-2) compared to the levels of vehicle-treated chondrocytes (Fig. 3). The mRNA levels of the other inflammatory TLR receptor, toll-like receptor-4 (TLR-4) were markedly lower in human articular chondrocytes compared to the TLR-2 mRNA levels (Fig. 3). In addition, TLR-4 mRNA levels were not increased by IL-1β treatment (Fig. 3). P15-1 or HMWHA treatment, when used separately, did not change the CD44 mRNA levels in IL-1β-treated cells. A mixture of P15-1 and HMWHA, however, significantly increased mRNA levels of CD44 in IL-1β-treated chondrocytes (Fig. 3). In addition, P15-1 alone, or mixed with HMWHA, decreased the mRNA levels of TLR-2 and TLR-4 in IL-1β-treated human articular chondrocytes (Fig. 3). HMWHA increased the mRNA levels of TLR-4, but did not affect the mRNA levels of TLR-2 in IL-1β-treated human articular chondrocytes (Fig. 3). These findings show that P15-1 decreases the mRNA levels of the inflammatory receptors TLR-2 and TLR-4 and, together with HMWHA, increases the mRNA levels of CD44 in IL-1β-treated human chondrocytes.

### P15-1 decreases prolonged activation of p38 MAPK in IL-1β-treated articular chondrocytes

**Figure 4:**
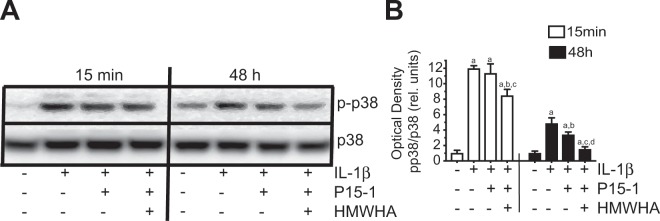
(**A)** Immunoblot analysis of cell extracts from human articular chondrocytes treated with IL-1β, IL-1β and P15-1, or IL-1β, P15-1 and HMWHA for 15 min or 48 h with antibodies specific for phosphorylated (activated) form of p38 (p-p38) or total p38 (p38). (**B)** The optical densities of the p-p38 and p38 bands were quantitated by densitometry. Results are expressed as optical density of p-p38 band divided by the optical density of the p38 band and relative units to the value of vehicle-treated cells at 15 min or 48 h, which was set as 1. The blot in (**A)** is representative of 3 separate experiments with similar results. The optical densities were analyzed on three immunoblots and values are mean ± SD.

Activation of NF-κB and p38 MAPK has been correlated with inflammation in a variety of cells, including articular chondrocytes^20,26,27^. Whereas NF-κB signaling in IL-1β-treated articular chondrocytes was not affected by P15-1 or combined P15-1/HMWHA treatment (data not shown), p38 MAPK activation after 15 min and 48 h treatment with IL-1β was reduced by combined P15-1/HMWHA treatment (Fig. 4A,B). The other two MAPK signaling pathways (ERK and JNK) were also not affected by P15-1 or P15-1 in combination with HMWHA (data not shown). As shown in Fig. 4, IL-1β treatment of human articular chondrocytes stimulated p38 MAPK signaling at 15 min and 48 h as indicated by the results of immunoblotting showing increased band intensity of the phosphorylated form of p38, whereas the amount of total p38 was not significantly different in vehicle-treated and IL-1β-treated cells (Fig. 4A,B). P15-1 alone reduced p38 MAPK signaling in IL-1β-treated chondrocytes as indicated by the reduced phosphorylated form of p38 in articular chondrocytes after 48 h treatment with IL-1β but not after 15 min treatment with IL-1β. P15-1 together with HMWHA inhibited the activation of p38 MAPK in chondrocytes treated with IL-1β for 15 min and to a greater degree in chondrocytes treated with IL-1β for 48 h than P15-1 alone (Fig. 4A,B). The degree of inhibition of p38 MAPK activity by P15-1 in the absence or presence of HMWHA was more pronounced in cells, which were treated for 48 h than in cells that were treated for 15 min with IL-1β (Fig. 4A,B). These findings show that P15-1, in synergy with HMWHA, reduces prolonged activation of p38 MAPK signaling in IL-1β-treated human articular chondrocytes.

### P15-1 together with HMWHA increases the thickness of the pericellular matrix (PCM) around articular chondrocytes

**Figure 5:**
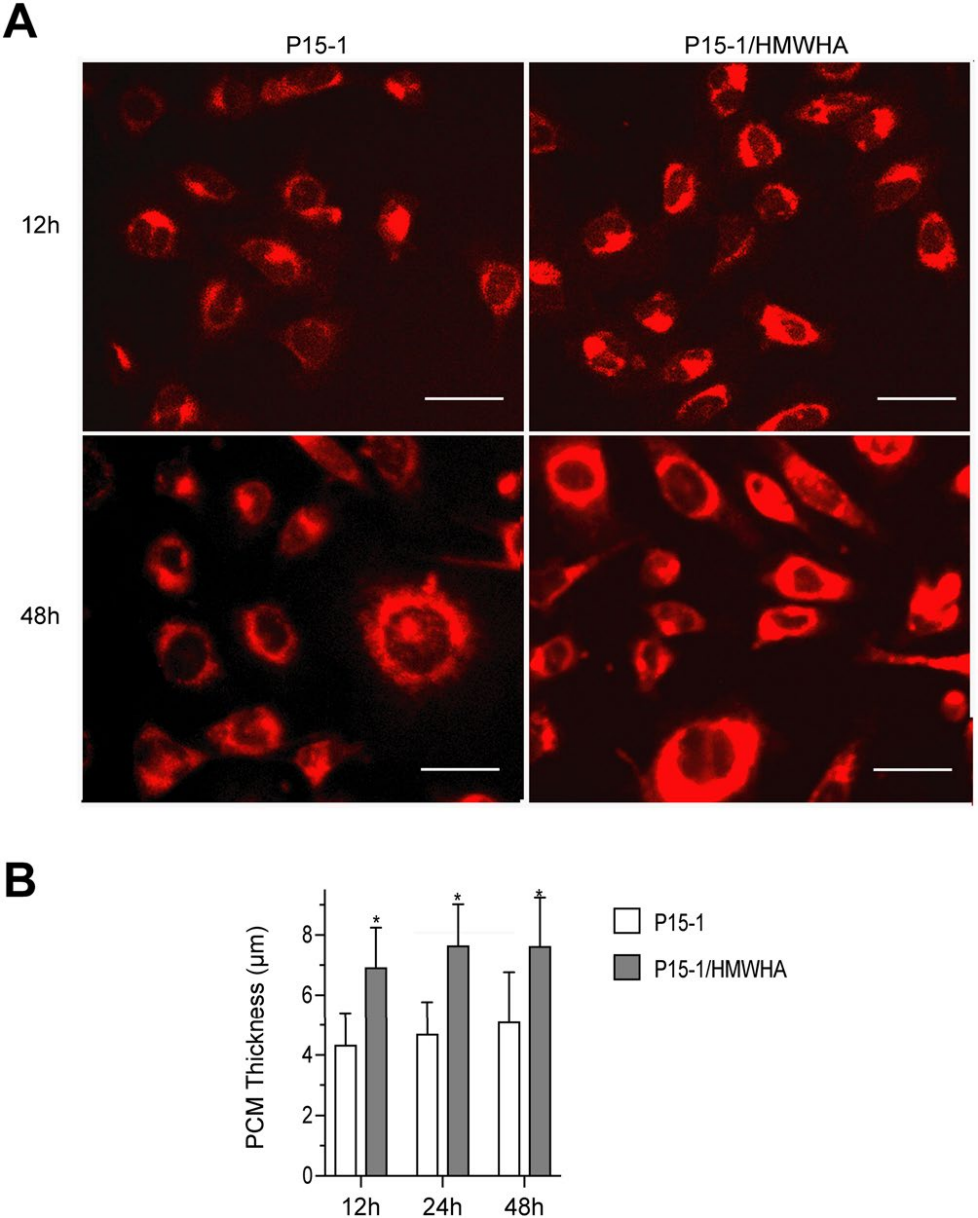
(**A)** Fluorescent microscopical images showing Cy5.5-labeled P15-1 located in the pericellular matrix (PCM) of human articular chondrocytes. Human articular chondrocytes were incubated for 12 h, 24 h and 48 h with Cy5.5-labeled P15-1 or Cy5.5-labeled P15-1 together with HMWHA. Fluorescent images shown in (**A)** were obtained after 12 h and 48 h incubation. (**B)** Average thickness of the HA fluorescent-labeled pericellular coat after 12 h, 24 h and 48 h incubation with Cy5.5-labeled P15-1 or Cy5.5-labeled P15-1 together with HMWHA. The average HA fluorescent-labeled pericellular coat was obtained by measuring the thickness of the HA coat that was fluorescent-labeled with Cy5.5- P15-1 of 15 chondrocytes in each treatment group. Data are shown as mean + SD. *p < 0.01.

To get first insights into the mechanism of how P15-1 together with HMWHA protects human articular chondrocytes, we cultured these cells in the presence of cyanine5.5 amine (Cy5.5)-labeled P15-1 (17 μg/ml) or a mixture of Cy5.5-labeled P15-1 (17 μg/ml) and HMWHA (1 mg/ml). After various time points (12 h, 24 h, 48 h) of incubation, cells were observed under a fluorescence microscope. The Cy5.5-labeled P15-1 accumulated in the PCM of articular chondrocytes most likely bound to HA (Fig. 5A). After 12 h, 24 h and 48 h the thickness of the fluorescent-labeled PCM increased in the presence of Cy5.5-labeled P15-1 together with HMWHA compared to the PCM in the presence of Cy5.5-labeled P15-1 alone (Fig. 5A,B).

### P15-1 decreases the inhibitory effect of conditioned media from IL-1β-treated human chondrocytes on chondrogenic differentiation of C3H10T1/2 cells

**Figure 6:**
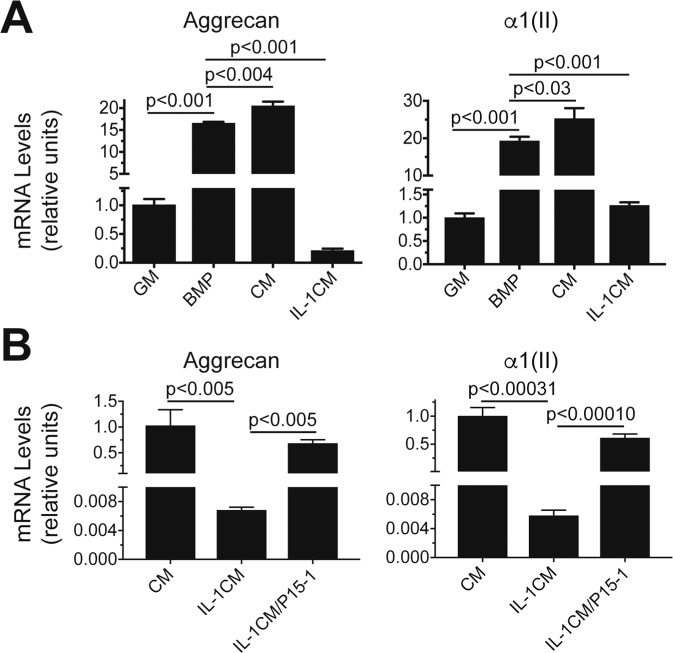
mRNA levels of aggrecan and type II collagen during chondrogenic differentiation of C3H10T1/2 cells in micromass cultures in the presence of conditioned medium from vehicle-treated, IL-1β-treated, or IL-1β/P15-1-treated human articular chondrocytes. (**A)** Micromasses were cultured in growth medium in the absence (GM) or presence of BMP-2 (BMP) for 6 days. In addition, micromasses were cultured in conditioned medium from vehicle-treated human articular chondrocytes and growth medium in a ratio of 1:1 in the presence of BMP-2 (CM), or conditioned medium from human articular chondrocytes treated for 24 h with IL-1β and growth medium in a ratio of 1:1 in the presence of BMP-2 (IL-1CM) for 6 days. (**B)** Micromasses were cultured in conditioned medium from vehicle-treated human articular chondrocytes and growth medium in a ratio of 1:1 in the presence of BMP-2 (CM), conditioned medium from human articular chondrocytes treated for 24 h with IL-1β and growth medium in a ratio of 1:1 in the presence of BMP-2 (IL-1CM), or conditioned medium from human articular chondrocytes treated for 24 h with IL-1β and P15-1 and growth medium in a ratio of 1:1 in the presence of BMP-2 (IL-1/P15-1CM) for 6 days. Levels of mRNA in (**A**,**B)** were determined by real-time PCR using SYBR Green and normalized to the level of 18S RNA. The mRNA levels are expressed relative to the levels of micromasses cultured in GM in (**A)** or relative to the levels of micromasses cultured in conditioned medium from vehicle-treated human articular chondrocytes and growth medium in a ratio of 1:1 in the presence of BMP-2 (CM) in (**B)**, which were set as 1. Data were obtained from triplicate PCRs using RNA from 3 different cultures. Values are the mean ± SD.

The mesenchymal stem cell line C3H10T1/2 differentiates into chondrocytes in the presence of bone morphogenetic protein-2 (BMP-2) in micromass culture^28^. Chondrogenic differentiation of these cells can be detected by markedly increased mRNA levels of aggrecan and type II collagen in BMP-2-treated micromass cultures compared to micromasses cultured in the absence of BMP-2 (Fig. 6A). When C3H10T1/2 micromasses were cultured in conditioned media from vehicle-treated human articular chondrocytes mixed with chondrogenic differentiation medium in a ratio of 1:1 in the presence of BMP-2 the mRNA levels of aggrecan and type II collagen were further increased compared to BMP-2-treated micromasses cultured in chondrogenic differentiation medium only (Fig. 6A). In contrast, micromasses cultured in conditioned media from IL-1β-treated human articular chondrocytes mixed with chondrogenic differentiation medium in a ratio of 1:1 in the presence of BMP-2 showed markedly reduced mRNA levels of aggrecan and type II collagen compared to BMP-2-treated micromasses cultured in chondrogenic differentiation medium only (Fig. 6A). When micromasses were cultured in conditioned medium from IL-1β and P15-1-treated human articular chondrocytes, the inhibitory effect on mRNA levels of aggrecan and type II collagen by conditioned medium from IL-1β-treated chondrocytes was reversed and the micromass cultures showed similar mRNA levels of aggrecan and type II collagen as micromasses cultured in chondrogenic differentiation medium in the presence of BMP-2 and conditioned medium from vehicle-treated chondrocytes (Fig. 6B). These findings suggest that reducing inflammation and catabolism in articular cartilage by P15-1 may ultimately help to promote chondrogenesis of precursor cells during cartilage repair.

## Discussion

This study shows that a 15-mer HA-binding peptide (P15-1) suppressed catabolic and inflammatory events in human articular chondrocytes treated with IL-1β, including the expression of catabolic and inflammatory factors (Cox-2, IL-6, iNOS), proteinases, such as MMP-13, and the inflammatory toll-like receptors TLR-2 and TLR-4. P15-1 decreased the prolonged activation of p38 MAPK signaling in IL-1β-treated human articular chondrocytes. p38 MAPK signaling has been correlated with inflammatory events in articular cartilage and inhibiting p38 MAPK signaling has been shown to reduce these inflammatory events^19,20,29^. Furthermore, our findings show that P15-1 was more effective in suppressing catabolic and inflammatory events in IL-1β-treated human articular chondrocytes when mixed with HMWHA. In addition, P15-1 and HMWHA alone reduced the mRNA levels of TSG-6 in IL-1β-treated chondrocytes. P15-1 in combination with HMWHA was more effective in suppressing TSG-6 expression. TSG-6 expression is being upregulated in many cell types during inflammation^25^. It has been shown to be one of the most significantly upregulated genes in the progression of OA^24,30^.

It has been suggested that HA affects inflammatory events in a variety of tissues in a size-dependent manner^31^. Low molecular weight (LMW)HA (e.g. molecular weight less than 200 kDa), which can be produced at sites of active tissue catabolism, was shown to promote inflammation via its effects on TLRs in a variety of cell types, including chondrocytes^31–33^. Specifically, it was shown that LMWHA can stimulate catabolic and inflammatory events in articular chondrocytes via its interaction with TLR-4, when that receptor is expressed^31–33^. Our current findings however, demonstrate that the expression levels of TLR-4 were much lower than the expression levels of TLR-2 in human articular chondrocytes. More importantly, IL-1β treatment increased the expression levels of TLR-2 but not TLR-4 in human articular chondrocyte cultures. In addition, another of our studies has shown increased amounts of HA released into the medium of IL-1β-treated human articular chondrocytes. The average molecular mass of the HA, however, remained high and fragmentation of HA was not detected^34^. In addition, the expression of RHAMM, a HA receptor that has been shown to stimulate inflammation in various tissues^35^, was downregulated in IL-1β-treated articular chondrocytes. These findings taken together suggest that HA fragments have not contributed to the stimulation of catabolic and inflammatory events in the IL-1β-treated human articular chondrocytes studied here.

In contrast to LMWHA, HMWHA is prevalent in uninjured tissues and has been shown to be anti-inflammatory based on its ability to cluster CD44 and inhibit pro-inflammatory CD44 and TLR signaling by LMWHA and other ligands^13,31,36–38^. In addition, other extracellular matrix components have been shown to influence HA integrity and its interactions with CD44. Especially, a group of HA-binding molecules, called hyaladherins, have been shown to alter the organization of HA and the nature and extent of cross-linked HA structures which may be essential for the anti-inflammatory properties of HMWHA^15,38^. For example, TSG-6 is responsible for the covalent transfer of heavy chains (HC) of inter-α-inhibitor (IαI) present in serum to HA^39^. HC-mediated crosslinking of HA may stabilize the pericellular matrix and inhibit inflammatory events. It can be speculated that P15-1 may enhance the pericellular HA network, whether directly or by affecting HA modification or binding of hyaladherins, resulting in a stabilized PCM, which protects the cell against pro-inflammatory and catabolic cytokine signaling. This mechanism may also explain why P15-1 together with exogenous HMWHA is more effective than P15-1 alone in reducing inflammatory and catabolic events in IL-1β-treated human articular chondrocytes, in which TSG-6 expression is highly upregulated (Fig. 2A). Future studies should be directed towards determining the exact mechanism of how P15-1 together with HMWHA inhibits inflammatory events in articular chondrocytes.

It is increasingly evident that inflammatory mechanisms play a central role in mediating the development and progression of OA^40^. In addition, a variety of inflammatory mediators are unleashed acutely in the joint after injury and may be responsible for the initiation of catabolic events in articular cartilage, including the expression of matrix degrading proteases, such as MMPs^41,42^. Furthermore, inflammatory events in the joint have been shown to inhibit chondrogenesis of MSCs, which for example is required for cartilage repair after microfracture surgery^43^. In this study we show that conditioned media from IL-1β-treated human articular chondrocytes inhibited chondrogenic differentiation of the MSC cell line C3H10T1/2. More importantly, conditioned medium from human articular chondrocytes treated with IL-1β and P15-1 reversed the inhibitory effect of conditioned medium from IL-1β-treated chondrocytes on chondrogenic differentiation of C3H10T1/2 cells. These findings suggest that the anti-inflammatory effect of P15-1 may be beneficial for the treatment of OA and for cartilage repair strategies that require the chondrogenic differentiation of MSCs in an inflammatory environment.

In conclusion, we have identified that a HA-binding 15-mer peptide (P15-1) shows anti-inflammatory effects on human articular chondrocytes cultured in the presence of IL-1β. Our findings suggest that the peptide inhibits inflammatory and catabolic events in human articular chondrocytes by reducing the prolonged activation of the p38 MAPK signaling pathway when cells are cultured in the presence of IL-1β. The p38 MAPK signaling pathway has been shown to play a major role in stimulating inflammatory and catabolic events in chondrocytes^20^. Our findings showing that P15-1 acts synergistically with HMWHA to further reduce inflammation in chondrocytes, suggest that the peptide may contribute to the cell-protective properties of HMWHA leading to greater CD44 clustering and the prevention of factors binding and signaling through inflammatory receptors, such as TLRs. Furthermore, our findings showing that medium from chondrocytes treated with IL-1β and P15-1 was less inhibitory on chondrogenic differentiation of C3H10T1/2 cells than medium from chondrocytes treated with IL-1β alone, suggest that reducing the inflammatory environment in the joint after injury or OA may enable MSCs to better repair cartilage lesions.

## Methods

### Ethics statement

Study protocols were approved by the Institutional Review Board of New York University School of Medicine (protocol #i-08-761). Written informed consent was obtained from all adult patients; minors (<18 years of age) were excluded from this study since there is no osteoarthritis in minors. All the methods were carried out in accordance with the approved guidelines.

#### Reagents

P15-1 (STMMSRSHKTRSHHV) was synthesized and purified using preparative high-performance liquid chromatography, and then characterized by electrospray ionization mass spectrometry (MS) Synthesis was carried out on Rink amide methylbenzhydrylamine resin (0.1 mmol) using automated (APEX 396 auto-synthesizer) solid phase peptide synthesis involving Fmoc deprotection and amino acid coupling cycles. Fmoc deprotection was carried out using 20% piperidine solution in N,N-dimethylformamide (DMF) throughout the synthesis. All amino acid couplings were carried out using four-fold excess of Fmoc-protected L-amino acid and 2-(1H-benzotriazol-1-yl)-1,1,3,3-tetramethyluronium hexafluorophosphate, 6 equivalents of N,N-diisopropylethylamine in DMF at 30 min and 90 min intervals. After each deprotection and coupling step, the resin was washed repeatedly with DMF and dichloromethane. The peptide was cleaved from the resin and all protecting groups were removed using a solution of 88% v/v trifluoroacetic acid, 5% v/v water, 5% m/v phenol, 2% v/v triisopropylsilane for 2-4 hours. The filtrate was collected, precipitated using cold tert-butyl methyl ether and pelleted via centrifugation at 3000 rpm at −50 °C for 10 minutes. Pellets were then dissolved in distilled-deionized water and lyophilized yielding a white powder. Analysis and purification of the peptide was performed using a 5-60% gradient solvent system consisting of 0.1% TFA in H_2_O (solvent A) and 0.1% TFA in acetonitrile (solvent B) at a linear flow rate of 1.5 mL/min and 20 mL/min for analytical and preparative High Perfomance Liquid Chromatography (HPLC), respectively. Analytical HPLC was performed using a Waters Sunfire reverse phase (RP)C18 column (4.6 mm × 150 μm, 5 μm), and preparative HPLC was performed using a Waters Sunfire OBD RP-C18 column (19.0 mm × 150 mm, 5 μm). Absorbance was detected at wavelengths of 220 nm and 254 nm using a Waters 2998 Photodiode Array detector. The collected fractions were then lyophilized to a solid, and subsequently analyzed by analytical Reversed-Phase High Performance Liquid Chromatography. (RP-HPLC). The peptide was purified by the above-mentioned protocol at a gradient of 10% to 45% with a retention time of 9.87 minutes (Suppl. Fig. 2). In addition, fractions were analyzed by electrospray ionization (ESI)-MS (Waters Micromass Quattro Micro^TM^ API) (Suppl. Fig. 3**)**. The MS spectra shows that the purity of the peptide used in this study was greater than 95% (Suppl. Fig. 3) HMWHA, as a 15 mg/ml sterile solution in physiological saline (Orthovisc, 2 ml per syringe) was purchased from DePuy Mitek, Inc. (Raynham, MA).

#### Cell culture

Human articular chondrocytes were isolated from articular cartilage samples obtained from patients undergoing total knee replacement surgery at NYU Langone Orthopedic Hospital. Articular cartilage tissue was obtained from female and male donors (donor age range 48–67). Sixty percent of patients undergoing knee replacement surgery at NYU Langone Orthopedic Hospital are female patients and 40% are male patients. In our study we did not observe age- or gender-dependent differences regarding cell responses to stimulation. Knee cartilage was harvested from regions with no macroscopically evident degeneration. The collection of tissue from patients undergoing knee replacement surgery was approved by the Institutional Regulatory Board at NYU School of Medicine. Human chondrocytes were isolated from these cartilage samples as described by us^44^. Cells were plated at density of 1.8 × 10^5^ cells/cm^2^ and grown in monolayer cultures in Dulbecco’s modified Eagle’s medium (DMEM; Life Technologies, Gaithersburg, MD) containing 10% fetal calf serum (FCS; HyClone, Logan, Utah), 2mM L-glutamine (Invitrogen, Carlsbad, CA), and 50 U/ml of penicillin and streptomycin (Invitrogen) (complete medium). These conditions have been shown to maintain their chondrocytic phenotype^45^. After cells have reached confluency, they were serum-starved for 24 h and then cultured in the presence of 10 ng/ml recombinant human IL-1β in phosphate-buffered saline (PBS) and 0.1% bovine serum albumin (BSA) for various time points. In addition, cells were treated with 17 µg/ml P15-1 in the absence or presence of 1 mg/ml HMWHA (Orthovisc). Control cells were cultured in the presence of vehicle (PBS/0.1% BSA). The weight ratio of HA to P15-1 used in this study was 60:1. This is the same as the ratio of 1 mg/ml HA to 17 µg/ml P15-1. This ratio was chosen such that each peptide would occupy only a small fraction of the available binding sites on HA. Since P15-1 is 15 amino acids long, the maximum peptide length is about 5 nm. On HA, 5 nm corresponds to 5 disaccharides. Therefore, each peptide P15-1 could occupy about 5 disaccharides on HA. We chose a ratio of about one P15-1 peptide per 75 binding sites on HA, or one P15-1 per 375 (75 × 5) disaccharides of HA. The formula weight for 375 disaccharides of HA is 375 × 400 Da = 150,000 Da. The formula weight of one P15-1 with 7 TFA is 2,580 Da. This is a HA: P15-1 weight ratio of 58:1, rounded to 60:1. This HA: P15-1 ratio turned out to be the best ratio to inhibit catabolic events in articular chondrocytes compared to other ratios (120:1, 30:1, 15:1) tested (data not shown).

The murine multipotential stem cell line, C3H10T1/2 clone 8, was obtained from American Type Culture Collection (ATCC; Manassas, VA). Monolayer cultures were maintained in DMEM containing 10% FCS (growth medium). To induce chondrogenesis, we used the miromass culture system, ITS + premix (insulin, transferrin, selenous acid, BSA and linoleic acid; Corning, Corning, NY) and bone morphogenetic protein (BMP)-2 using 10 µl drops of cells at 1 × 10^7^ cells/ml as described previously^28^. Micromass cultures were maintained in growth medium with or without 100 ng/ml recombinant murine BMP-2 (Peprotech, Rocky Hill, NJ) for up to 6 days. Medium was changed every other day. Micromass cultures were also maintained in growth medium and conditioned medium from vehicle-treated human articular chondrocytes, or from human articular chondrocytes cultured for 24 h in the presence of IL-1β, or IL-1β and P15-1 in a ratio of 1:1 in the presence of BMP-2.

#### Fluorescence microscopy

After incubation of human articular chondrocyte cultures with Cy5.5-labeled P15-1 alone or together with 1 mg/ml HMWHA for 12 h, 24 h and 48 h, chondrocyte cultures were observed under fluorescence microscopy using an Olympus microscope. Images were taken using camera. The width of the fluorescent-labeled PCM was measured using software. The mean ± SD of the measurements of the width of the PCM of 15 different cells was recorded.

#### Reverse transcription–polymerase chain reaction (PCR) and real-time PCR analysis

Total RNA was isolated from chondrocyte cultures using an RNeasy Mini kit (Qiagen, Valencia, CA). Levels of messenger RNA (mRNA) for aggrecan, CD44, IL-6, iNOS, MMP-13, TLR-2, TLR-4, TSG-6, and type II collagen were quantified by real-time PCR as previously described^46^. Briefly, 1 µg of total RNA was reverse transcribed using the High Capacity cDNA synthesis kit (Applied Biosystems, Foster City, CA). A 1:100 dilution of the resulting cDNA was used as the template to quantify the relative content of mRNA by real-time PCR (ABI Stepone Plus; Applied Biosystems), using the appropriate primers and RT^2^ SYBR Green ROX FAST Mastermix (Qiagen). PCRs were performed at 95 °C for 10 minutes, followed by 40 cycles of 95 °C for 10 seconds and 60 °C for 30 sec, and 1 cycle of 95 °C for 15 seconds and 60 °C for 1 minute. The 18S RNA was amplified at the same time and used as an internal control. The cycle threshold values for 18S RNA and the samples were measured and calculated. Transcript levels were calculated according to the equation x = 2^−ΔCq^, where ΔCq = Cq_exp_ − Cq_18S_, or relative transcript levels were calculated as x = 2^−ΔΔCt^, in which ΔΔCt = ΔE − ΔC, ΔE = Ct_exp_ − Ct_18S,_ and ΔC = Ct_ctl_ − Ct_18S_.

#### Sodium dodecyl sulfate (SDS)-polyacrylamide gel electrophoresis and immunoblotting

Whole cell lysates were collected from cultured chondrocytes using ice-cold RIPA buffer (Pierce) containing a mixture of protease inhibitors (Pierce) and phosphatase inhibitors (Pierce, Rockford, IL). To determine the amount of phosphorylated and total p38, cell lysates were dissolved in 4 x NuPAGE SDS sample buffer containing a reducing agent (Invitrogen), denatured at 70 °C for 10 min, and analyzed by electrophoresis in 10% bis-Tris polyacrylamide gels. Samples were electroblotted onto nitrocellulose filters after electrophoresis. After blocking with a solution of low-fat milk protein, blotted proteins were immunostained with primary antibodies specific for phosphorylated p38 or total p38 (Cell Signaling Technology, Danvers, MA) and then peroxidase-conjugated secondary antibody. The signal was detected by enhanced chemiluminescence (Pierce) as previously described^46^.

#### Statistical analysis

Data was analyzed using PRISM statistical analysis software. Descriptive statistics (mean, SD, medians, and interquartile ranges) were generated for each outcome variable. The descriptive statistics was generated for each group separately. Analysis of the variance (ANOVA) was used to assess differences in means among 3 or more groups, while comparisons of two groups was made using t tests. If there were significant differences in ANOVA, pairwise tests were conducted to assess specific differences using Tukey’s multiple comparison procedure. A p-value < 0.05 was used as a threshold for statistical significance.

## Supplementary information

Supplementary information
