# Supplementary material for: A Hyaluronan-binding Peptide (P15-1) Reduces Inflammatory and Catabolic Events in IL-1β-treated Human Articular Chondrocytes
Source: Sci Rep. 2020 Jan 29;10:1441. doi: 10.1038/s41598-020-57586-7 (PMC6989647; doi:10.1038/s41598-020-57586-7)
Supplement: Supplementary file 1 — Supplementary information [file 41598_2020_57586_MOESM1_ESM.pdf]

Supplemental Material

A Hyaluronan-binding Peptide (P15-1) Reduces Inflammatory and Catabolic Events in IL-1β-treated Human Articular Chondrocytes

Claire Shortt[a,b], Leonard G. Luyt[c,d], Eva A. Turley[c,d], Mary K. Cowman[a,e], and Thorsten Kirsch,[a,e]

[a]Musculoskeletal Research Center, Department of Orthopaedic Surgery, New York University School of Medicine, New York, NY, USA

[b] The University of Western Ontario, London, ON, Canada

[c] London Regional Cancer Program, Lawson Health Research Institute, London, ON, Canada

[d] Department of Biomedical Engineering, New York University Tandon School of Engineering, New York, NY, USA

[b]Current address: FoodMarble Digestive Health, Dublin 2, Ireland

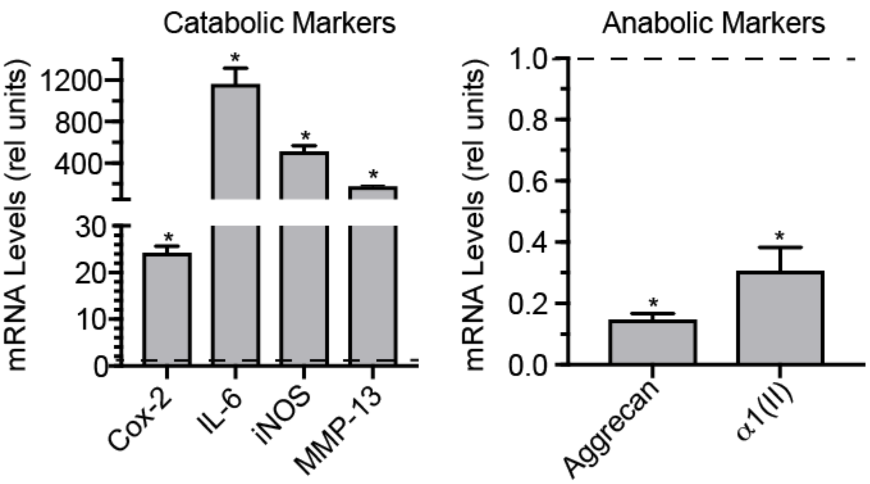

Suppl. Figure 1: mRNA levels of catabolic markers and anabolic markers in human articular chondrocytes cultured in the absence or presence of IL-1β. Human articular chondrocytes after reaching confluency were serum-starved for 24 h followed by treatment with IL-1β in PBS/0.1% BSA or PBS/0.1% BSA for 24 h. mRNA levels of catabolic markers (Cox-2, IL-6, iNOS, MMP-13), articular cartilage markers (aggrecan, type II collagen (α1(II)) were determined by real-time PCR using SYBR Green and normalized to the level of 18S RNA. The mRNA levels are expressed relative to the levels of vehicle-treated cells, which were set as 1 (dotted line). Data were obtained from triplicate PCRs using RNA from 3 different cultures. Values are the mean ± SD. *$p < 0.01$ vs. vehicle-treated cells.

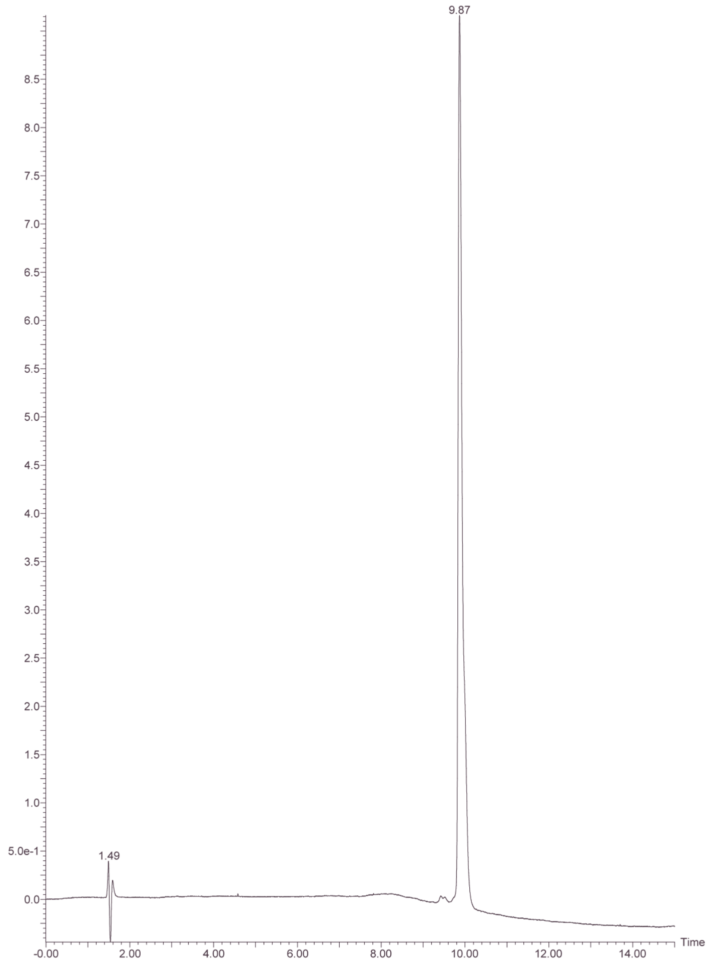

Suppl. Figure 2: UV Chromatogram of the P15-1 fraction analyzed by analytical Reversed-Phase High Performance Liquid Chromatography (RP-HPLC).

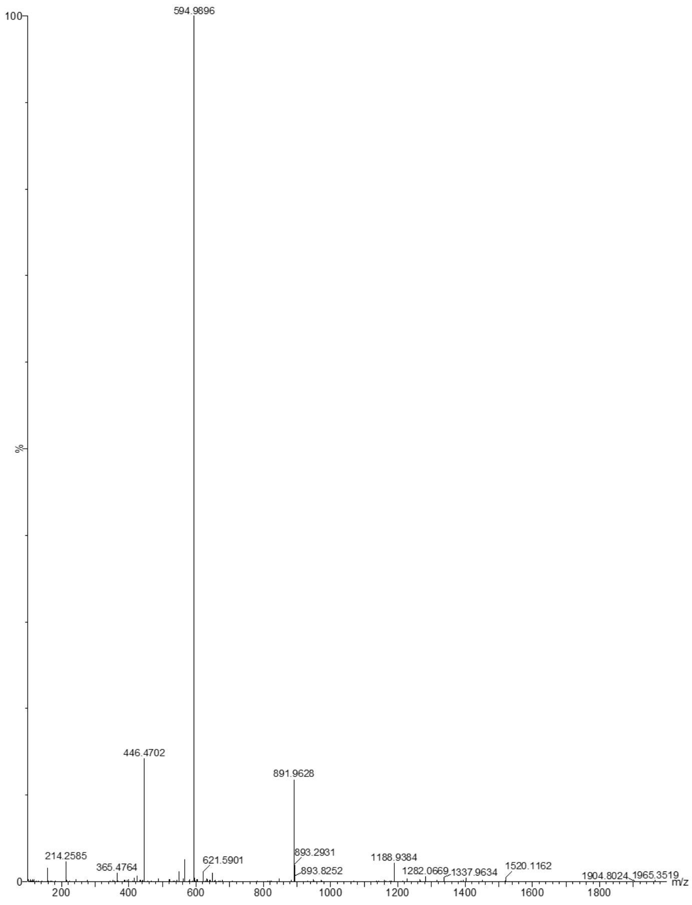

Suppl. Figure 3: MS Spectra of P15-1. Purity >95%. HRMS (ESI+): m/z calculated for $C_{71}H_{120}N_{28}O_{22}S_2$, Calculated $[M+2H]^{+2}$ = 891.9378; Found $[M+2H]^{+2}$ = 891.9628, Calculated $[M+3H]^{+3}$ = 594.6270; Found $[M+3H]^{+3}$ = 594.9896.

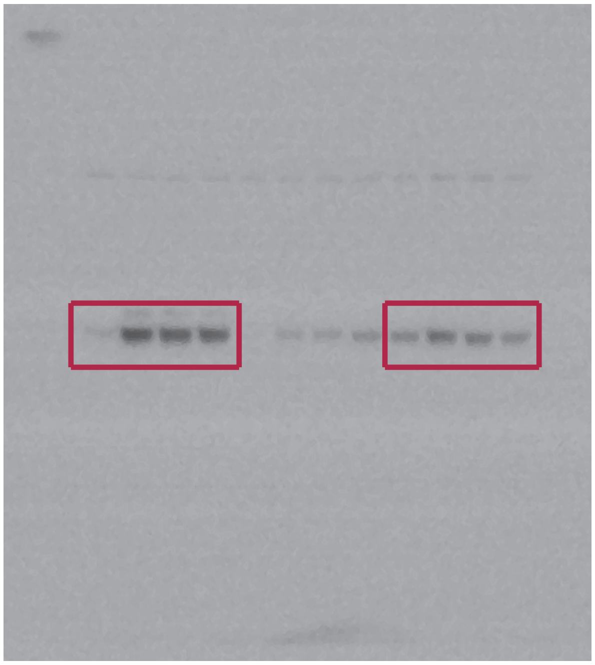

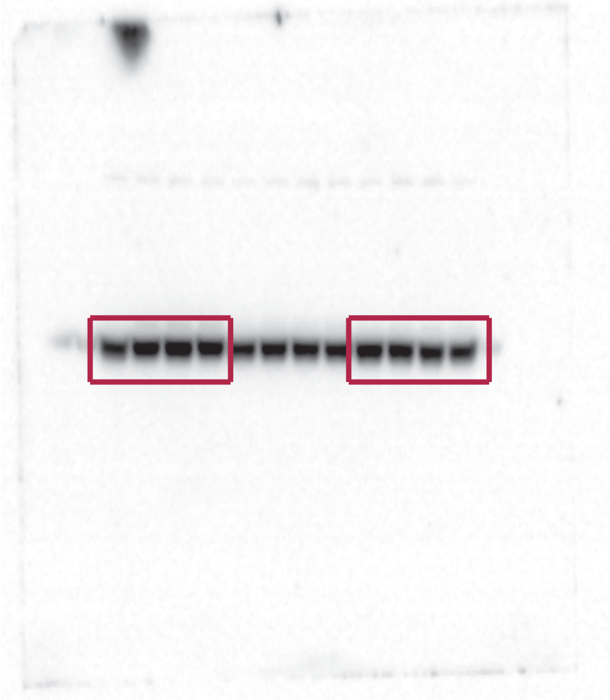

Supplementary Figure 4.

Full-lengths blots used to generate Figure 3A.
